# Self-Supervised Learning to Detect Key Frames in Videos

**DOI:** 10.3390/s20236941

**Published:** 2020-12-04

**Authors:** Xiang Yan, Syed Zulqarnain Gilani, Mingtao Feng, Liang Zhang, Hanlin Qin, Ajmal Mian

**Affiliations:** 1 School of Physics and Optoelectronic Engineering, Xidian University, Xi’an 710071, China; xyan@xidian.edu.cn (X.Y.); hlqin@mail.xidian.edu.cn (H.Q.); 2 School of Science, Edith Cowan University, Joondalup 6027, Australia; 3 School of Computer Science and Technology, Xidian University, Xi’an 710071, China; mtfeng@xidian.edu.cn (M.F.); liangzhang@xidian.edu.cn (L.Z.); 4 Computer Science and Software Engineering, University of Western Australia, Crawley 6009, Australia; ajmal.mian@uwa.edu.au

**Keywords:** key frames, self-supervised learning, convolutional networks, two-stream ConvNets

## Abstract

Detecting key frames in videos is a common problem in many applications such as video classification, action recognition and video summarization. These tasks can be performed more efficiently using only a handful of key frames rather than the full video. Existing key frame detection approaches are mostly designed for supervised learning and require manual labelling of key frames in a large corpus of training data to train the models. Labelling requires human annotators from different backgrounds to annotate key frames in videos which is not only expensive and time consuming but also prone to subjective errors and inconsistencies between the labelers. To overcome these problems, we propose an automatic self-supervised method for detecting key frames in a video. Our method comprises a two-stream ConvNet and a novel automatic annotation architecture able to reliably annotate key frames in a video for self-supervised learning of the ConvNet. The proposed ConvNet learns deep appearance and motion features to detect frames that are unique. The trained network is then able to detect key frames in test videos. Extensive experiments on UCF101 human action and video summarization VSUMM datasets demonstrates the effectiveness of our proposed method.

## 1. Introduction

Videos normally contain 30 frames per second and more information than is actually required for many computer vision tasks. Typical applications of key frame detection are video summarization, action recognition and visual simultaneous localization and mapping. To process all frames requires extensive memory and computational resources. In many applications, a few and sometimes even one, key frames may be sufficient to achieve the desired results e.g., we can recognize some actions from one frame. Similarly, video summarization itself is the task of finding key frames in a video to summarize the entire video content.

In this paper, we address the problem of automatically annotating and detecting key frames in a video. A video is represented as a sequence of continuous frames and the aim is to automatically annotate a set of frames of interest. Here we define “interest” is an abstract concept that denotes the frames can be representative of the video content, meanwhile, diverse to reduce the redundancy [1]. We assume there are training videos without annotated key frames, and our goal is to train a deep neural network that can automatically annotate key frames in training videos. Efficient key frame extraction modules have also enabled a boost in performance of other high-level visual tasks, such as video summarization [1,2,3,4,5,6,7,8], action recognition [9,10,11], video retrieval [12], visual simultaneous localization and mapping (SLAM) [13,14,15], video annotation [16] and realistic videos generation [17]. For human action recognition, the key frames are the ones that can well represent the whole action. For video summarization, the key frames are the set of frames that summarize the video. For SLAM, key frames record the most representative geometry maps or landmarks (dense depth, pose) among its neighbouring frames. For video generation, the key frames are the first and last frames of the video clip to be generated, respectively.

A typical approach to address this problem is to invite human subjects to watch a video and annotate key frames. Due to the inherent subjectivity of the task, it is not possible to obtain a consensus on the key frames. The videos with labelled key frames can then be used to learn models for automatic detection of key frames in previously unseen videos. In this case, the annotated key frames are treated as gold standard. While the users are often well instructed on the annotation task, discrepancies are still expected due to many uncontrollable individual factors such as idiosyncratic viewing preferences and whether the person was attentive etc. Another approach is to use deep neural networks which can deliver state-of-art performance for many visual tasks including key frame detection. However, deep models require large datasets for training, which are time-consuming to annotate by humans.

To push forward on high-level analysis and understanding of various tasks in videos, we argue that the development of automatic key frame detection is invaluable. To this end, we propose a deep self-supervised two-stream ConvNet for automatically annotating key frames in a video. We particularly target the case of analyzing human action videos to annotate key frames. Our work is based on the two-stream convolutional network architecture [18]. This architecture consists of appearance and temporal streams. The appearance stream extracts spatial features relevant to the scenes and objects depicted in the video, while the temporal stream extracts temporal features such as movement of the observer (the camera) or the objects. The appearance stream ConvNet takes a single video frame and outputs the corresponding appearance feature. The temporal stream ConvNet takes the optical flow image of the corresponding temporal frame in a video and outputs the temporal deep feature. Each stream is implemented using a deep ConvNet. To well represent video information, the two stream features are first fused and then the fused features of each video frame are used to output a score for the frame. Hence every frame in a video is given a score. Finally, a smooth fitting function is applied to fit a key frame curve to the frame wise outputs. Peaks in this curve correspond to highly discriminant frames which are regarded as key frames.

**Figure 1:**
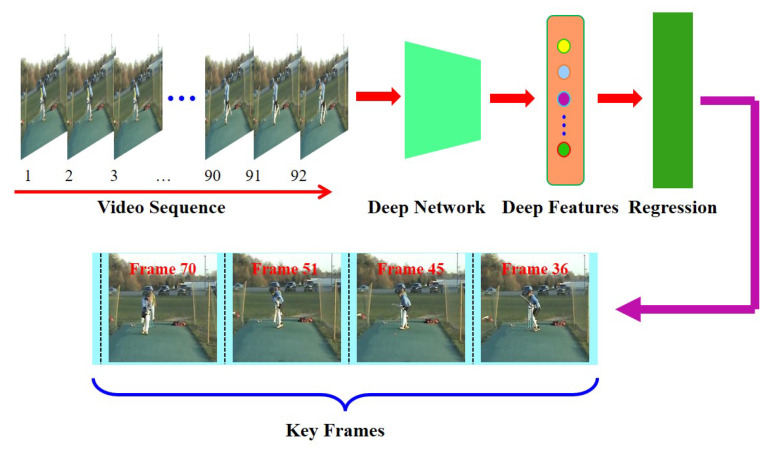
**Conceptual overview of our approach**. The proposed method automatically annotates key frames as the discriminant frames in a video to avoid the time-consuming and subjective manual labelling. Discrimnant analysis is performed on the framewise features extracted using a pretrained CNN. This example is of “Cricket Shot” with 92 frames. Our method marks four frames as key frames. Note that these four key frames can still describe the class of the human action.

To train the proposed automatic deep key frame detection network, we require a large labeled dataset of videos and their corresponding keyframes. To address this problem, we draw support from the powerful feature representation ability of Convolutional Neural Networks (CNNs) for images and the class separation and dimensionality reduction ability of Linear Discriminant Analysis (LDA) [19]. We devise a novel automatic labelling strategy instead of manual annotation for training the deep key frame detection model. Since there is no specialized dataset for key frame annotation research, we use the benchmark human action dataset UCF101 [20] and video summarization dataset VSUMM [21] to perform our experiments. Our key frame annotation model takes a long human action video as input, and outputs the key frames of an action instance, which can well represent the corresponding action instance, as shown in Figure 1. Our contributions are summarized as follows: (1) We propose a novel strategy for automatic labelling/annotation for discriminant (key) frames in training videos. (2) We introduce a novel two-stream ConvNet that is trained in a self-supervised manner, using labels generated from the previous method, to detect key frames in videos in real time. (3) We perform experiments on a popular human action recognition dataset UCF101 to show the efficacy of our method for automatic detection of key frames. To verify how well our network generalizes to other datasets, we perform key frame detection on the popular video summarization dataset VSUMM using our model trained on UCF101.

## 2. Related Work

Existing key frame annotation approaches for various applications rely on learning from manually annotated data. For example, the most popular key frame based video summarization approaches leverage the key frames that are annotated by several human subjects from different backgrounds. To the best of our knowledge, there is no unsupervised learning method for automatic detection of key frames in videos. Instead, the bulk of related works in a broader sense comes from video annotation and key frame detection.

### 2.1. Video Annotation Methods

The process of ground truth annotation has become a fundamental task in the development of machine learning based computer vision applications. Due to its time-consuming nature, various tools and strategies have emerged to facilitate the annotation task. For object detection in videos and related tasks, many popular annotation tools have been exploited such as ViPER [22], LabelMe [23] etc. Moreover, various mathematical methods have also been proposed to perform the annotation task. These can be divided into three categories [24]: probability and statistics [25], graphs [26,27] and machine learning [6,24,28,29,30,31] based methods. Nevertheless, for the closely related annotation tasks such as action detection, action proposals, action recognition and activity understanding, many video datasets exist. For example, the most influential action datasets ActivityNet [32], Hollywood [33], Charades-STA [34] provide ground truth action labels. Most of these labelling methods are interactive and require a human-in-the-loop approach. For example the ActivityNet [32], which is a large-scale video benchmark for human activity understanding, used multiple Amazon Mechanical Turk workers to annotate each video with the temporal boundaries associated with the activity. Some of these methods can assist the users during the annotation process at the frame-based or sequence-based level. The key frame based video summariztion problem is the closest to our key frame annotation task. For that research, there are some popular datasets such as Youtube dataset [21], SumMe [35] and TVSum [36]. In most of these datasets, each video is annotated by multiple human users to ensure correctness of the annotations.

### 2.2. Key Frame Detection

**Conventional Methods:** Many earlier approaches in this domain rely on using a segmentation based pipline. Such methods typically extract optical flow and SIFT features [9,37,38,39]. One of the first works [9,37] describe a video with optical flow and detect local minimum changes in terms of similarity between successive frames. Later works improved upon this pipline by using keypoints detection for feature extraction [38,39], extracting local features via a SIFT descriptor and pooling the keypoints to find key frames in videos. All of these approaches have a common limitation that they may extract key frames that are redundant rather than fully cover the video content.

Another class of conventional methods rely on clustering the features, such as color histograms, of video frames. These methods determine the key frames in a video by detecting a representative frame from each cluster e.g., the frame whose features are nearest to the cluster mean. Zhuang et al. [40] proposed an unsupervised clustering method for identifying the key frame that takes into account the visual content and motion analysis. Cernekova et al. [41] improved upon the method of Cernekova et al. [42] using the mutual information (MI) and the joint entropy (JE) between consecutive video frames. Using the MI to measure information transported from one frame to another, and JE to exploit the inter-frame information flow respectively, they were able to detect key frames. Tang et al. [43] proposed a key frame extraction approach based on image entropy and density clustering for hand gesture recognition. Vazquez et al. [44] proposed a spectral clustering based key frame detection method that builds a graph to capture a feature’s locality in a video sequence instead of relying on a similarity measure computed by the features shared between two images. It is worth mentioning here the works on sparse dictionary and Multiple Instance Learning (MIL) [45,46,47,48,49]. These methods use dictionary learning or the MIL framework to learn features from video frames and subsequently to detect the key frames for video summarization, action recognition or video event detection.

**Deep Learning Methods:** To overcome the limitations of conventional methods, more recent works focus on designing deep learning models to tackle the problem of key frame detection. Several supervised and unsupervised models have been proposed for key frame detection in videos which significantly boost the performance of various downstream tasks [1,4,14,17,49,50,51,52,53,54,55,56]. Yang et al. [50] first introduced the bidirectional long short term memory (Bi-LSTM) for automatically extracting the highlights (key frames) from videos. Several deep learning based methods for key frame detection in videos have been proposed [4,51]. Mahasseni et al. [4] first apply the Generative Adversarial Networks (GAN) to key frame detection in videos, which uses CNNs to extract the feature of each frame and then encodes the feature via LSTM. Kar et al. [51] adopted two-stream CNNs containing spatial and temporal networks with the MIL framework to detect the key frames with high scores in videos for action recognition. Huang et al. [5] proposed a novel key frame selection framework for comprehensive video summarization. Their method introduced a self-attention model to select key frames sequences inside shots. Jian et al. [57] also proposed a deep key frame extraction method for sports training via estimating the pose probability of each frame and the difference of neighboring probability. Wen et al. [17] introduced a key frame based video generation approach. This approach takes advantage of key frames in videos and GANs to obtain realistic videos. Moreover, Sheng et al. [14] proposed an unsupervised collaborative learning of key frame detection and visual odometry towards monocular deep SLAM, significantly improving the performance of SLAM.

**Figure 2:**
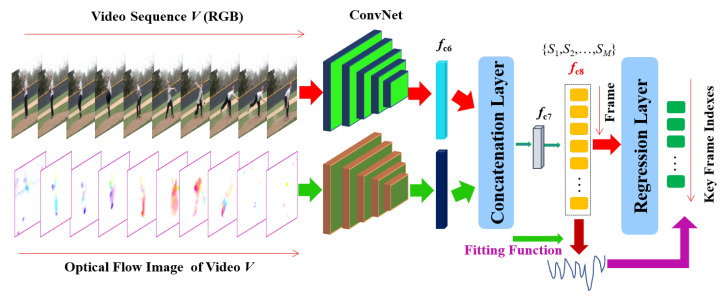
An overview of the deep key frame detection framework. The appearance network operates on RGB frames, while the motion network operates on the optical flow represented as images. The feature maps from the appearance and motion ConvNets are aggregated to form a spatio-temporal feature representation.

Given a test video, our goal is to devise an effective strategy for annotating key frames in the video and subsequently develop a key frame detection algorithm. To this end, we introduce a deep regression ConvNet with two-streams to learn and fuse both spatial and temporal features for exploiting spatio-temporal information in videos. Spatio-temporal features have been demonstrated to be very effective in video based classification and recognition tasks in the computer vision field [58,59,60,61,62,63]. The proposed network model requires labelled key frames in training videos to learn the desired network parameters. As mentioned before, key frame annotation is a laborious and time consuming task. To address this problem, we use Linear Discriminant Analysis to distinguish key frames from others and obtain the required labels in an unsupervised way for the training videos. Next, we used this labelled data to train the two stream ConvNet to regress input frames over the LDA scores. In this section, we first introduce our proposed key frame automatic annotation framework as shown in Figure 2. Next, we describe the proposed two stream deep ConvNet model for key frame detection in detail.

### 2.3. Problem Formulation

Key frame detection is a pre-requisite for video summarization and other video understanding tasks. These methods represent a video containing a large number of sequential frames by only a few (key) frames which can still well represent the original video. If we could obtain the key frame annotations (labels) for a training set of videos, these could be used to learn various models to perform the above tasks. Let video *V* be represented as a collection of frames $\left(y_{1},y_{2},\cdots ,y_{T}\right)$, where *T* is the total number of frames in video *V* and $y_{t}$ is the *i*-th frame. Hence, the key frame annotation set $Y_{kf}$ is defined as follows:

(1) $$Y_{kf}=S_{kf}\left(V\right)$$

where $S_{kf}$ is a function that automatically annotates key frames.

In this paper, we propose a self supervised method that learns to automatically detect key frames in videos. The proposed method has two main parts. In the first part, frame-level video labelling is performed using the Linear Discriminant Analysis (LDA). Spatial and temporal features are extracted using two CNNs and LDA is used to find a projection that minimizes the distance between same action videos and maximizes the distance between different action videos. We use a human action video dataset for this where the action labels are provided. After projection on the LDA space, the distance of a frame from the class mean is used as a uniqueness measure for the frame. The more unique a frame is, the higher the chances that it is a key frame. In the second step, a two stream CNN is trained to regress video frames on the uniqueness score obtained from the LDA projection of the first step. Once the CNN is trained, it is ready to output the uniqueness score of frames in a video and the frames corresponding to the peaks are selected as key frames.

### 2.4. Frame-Level Video Labelling

To learn our supervised key frame automatic annotation model, we need the ground-truth (labelled key frames) in videos. Since manual labelling is expensive, we use an alternate approach to obtain labels automatically. We first obtain the frame-level features by fusing the video’s appearance and motion information. Appearance features are extracted with a pretrained CNN which outputs a feature vector of an RGB frame. Motion features are obtained from optical flow. Optical flow output is a two dimensional vector for each pixel. Using the direction of the vector and a circular 2D colormap, we convert each directional vectors to an RGB pixel. Hence, the optical flow output becomes a pseudo RGB image which is also passed through a pretrained CNN to get a feature vector.

**Figure 3:**
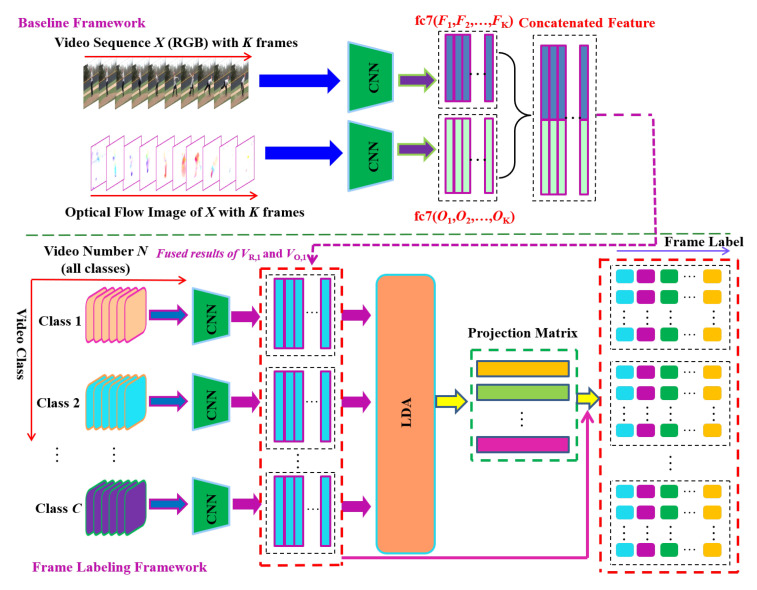
**Block Diagram.** Automatically generating labels to train the deep key frame automatic annotation framework. The appearance and motion information (the output of fc7 layer of VGG-16 [58]) are concatenated as a spatio-temporal feature vector. Thereon, LDA is applied to all the feature vectors of all the classes of training videos to project the feature vectors to a low dimensional feature space (LDA space) and obtain the projection vectors of each class videos. Finally, the projection vectors are used to calculate the frame-level video labels.

The process of frame-level video labelling is illustrated in Figure 3. We denote a video as $X=\left[x_{1},x_{2},\cdots ,x_{K}\right]$, $x_{k}\in R^{224\times 224\times 3}$, with each frame $x_{k}$ represented as RGB image. The input video resolution of 224×224 has been selected as it is in line with the required input resolution of VGG-16 model which we use in key frame detection (See Section 3.1). Assume a video *X* belonging to labelled class *c*, $c\in \left\{1,2,\cdots ,C\right\}$, the output feature map of the RGB frame from a CNN at a fully connected layer $\left(F_{1},F_{2},\cdots ,F_{K}\right)$, and its output feature map of the corresponding optical flow image from a CNN at the same fully connected layer is $\left(O_{1},O_{2},\cdots ,O_{K}\right)$. $F_{k}$ and $O_{k}$ are the CNN features of *k*-th RGB frame and its corresponding optical flow image. Similarly, we can obtain the RGB CNN features and the corresponding optical flow image CNN features of each training video $\left(V_{R,1},V_{R,2},\cdots ,V_{R,N}\right)$ and $\left(V_{O,1},V_{O,2},\cdots ,V_{O,N}\right)$, where *N* denotes the number of training videos, $V_{R,n}$ and $V_{O,n}$ represent RGB CNN features and its corresponding optical flow CNN features for *n*-th training video. Specifically, $V_{R,n}$ and $V_{O,n}$ are composed of $\left(F_{1},F_{2},\cdots ,F_{K}\right)$ and $\left(O_{1},O_{2},\cdots ,O_{K}\right)$, respectively.

## 3. Proposed Approach

The appearance and motion feature vectors of each frame are fused to form a combined representation enhancing the frame representation capability. The fused features can be written as $\left(V_{F1},V_{F2},\cdots ,V_{FN}\right)$. For self-supervision we define key frames as those which are very different in their appearance and optical flow features from the rest of the frames. To determine how different a given frame in a video is, we adopt the Linear Discriminant Analysis (LDA) [64] technique to discriminate between videos from different classes. More precisely, we aggregate the fused features of all the videos belonging to the same class *A* as a matrix $V_{A}$, and all the features of video from other classes as $\left\{V_{1},V_{2},\cdots ,\right\}$. Next, we use LDA to learn *C* projection matrices. For each class, its LDA projection matrix minimizes the within class distance (class *A*) and maximizes the between class distance (class *B*).

(2) $$V_{A}=V_{1}$$

(3) $$V_{B}=\left[V_{2},V_{3},\cdots ,V_{C}\right]$$

Given $V_{A}$ and $V_{B}$, we can perform LDA to obtain the projection vector $W_{A}$,

(4) $$W_{A}=\mathrm{LDA}\left(V_{A},V_{B}\right)$$

Then we use $W_{A}$ to calculate each frame score (label value) for each training video of class *A*:

(5) $$f_{i,m}={∥F_{i,m}-W_{A}W_{A}^{T}F_{i,m}∥}_{2}$$

where $F_{i,m}$ represents the feature vector of the *i*-th frame of the *m*-th video of class *A* and $f_{i,m}$ is essentially a score for the frame on how different it is. The above is essentially a one-vs-all two class LDA process, which is repeated for each class. For example, if there are C=20 classes, we will repeat the process for each of the 20 classes to obtain the feature score of each frame in each video of each class for all the training videos via Equation (5).

### 3.1. Learning a Deep Model for Automatic Key Frame Detection

**Figure 4:**
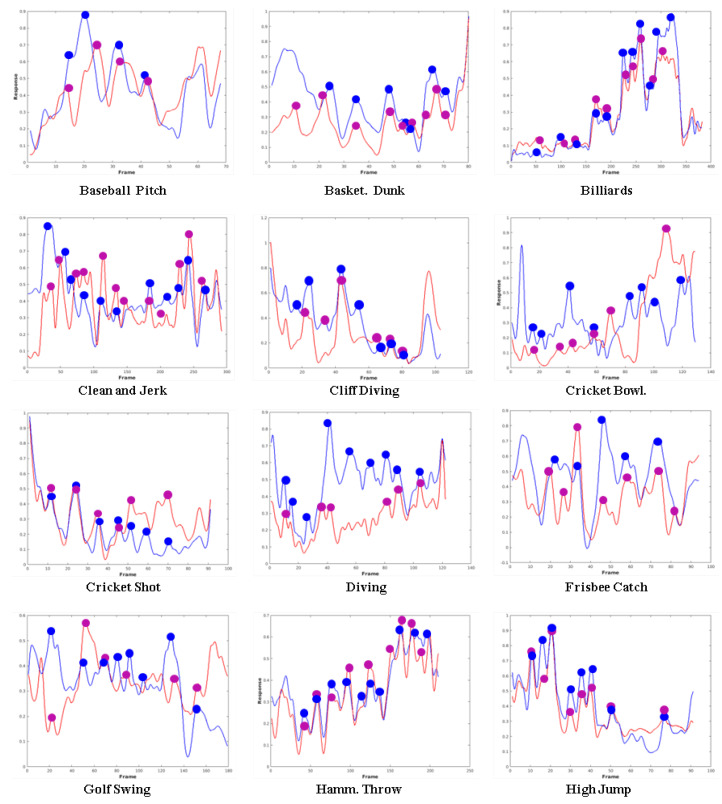

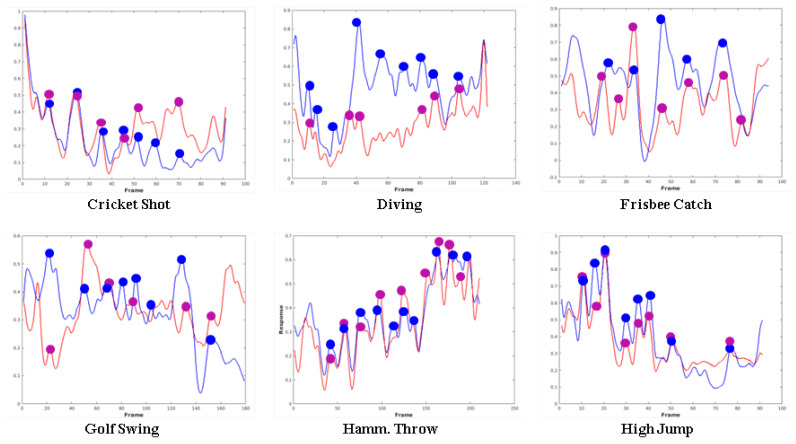
Key frame labelling score results and their respective automatically annotated ground truth. The blue curve represents the ground truth annotation scores while the red curve represents the labelling results from our two-stream network. The temporal location of ground truth and detected key frames are shown in blue and purple solid circles respectively.

Now that we have a label $f_{i,m}$ for each frame in each training video obtained above, learning a deep model to replicate the same output becomes straight forward. Since $f_{i,m}$ is a continuous value rather than a discrete value, we will need to learn a regression network rather than a classification network. The appearance and optical flow features of the training videos are again passed through a two stream CNN and the output features are concatenated in a similar way as above. Our proposed model is a deep two-stream convolutional neural network where one stream is appearance stream $S_{1}$ and the other is motion (optical flow) stream $S_{2}$. Note that these two streams have the same network architecture which is similar to the VGG-16 network [58]. The major difference is that we remove the last layers (fc7, fc8, and softmax) from the second fully connected layer (fc6) onwards. For convenience, we denote the fully connected layer fc6 from appearance and motion streams as fc6-1 and fc6-2. The input to the new fully connected layer of our deep two-stream ConvNet is formed by concatenating fc6-1 and fc6-2 layer outputs and then followed by new fc7 and fc8 fully connected layers. The final layer is a regression layer to compute Euclidean loss from the ground truth $f_{i,m}$. Since, $f_{i,m}$ is computed automatically, we call our method self-supervised as no manual supervision is required.An overview of our two-stream network is shown in Figure 4. Note that we use a single loss to train our two stream network from end-to-end.

To detect the key frames, an input video *V* is passed through the fully trained network described above and the prediction outputs are denoted as $\left\{S_{1},S_{2},\cdots ,S_{M}\right\}$. Next, we fit a curve to the outputs $\left\{S_{1},S_{2},\cdots ,S_{M}\right\}$ using a *smooth spline* function. From Equation (5), it is evident that the frame label or score $f_{i,m}$ represents the similarity of that frame with other frames of the same category. A low score corresponds to a small change between frames which suggest that the action in that particular frame is not significant. However, a large $f_{i,m}$ score, or a maximum between two minima in the fitted curve denotes sudden transition and might point to a significant activity. We annotate the local maximum between any two minima in the fitted curve of a certain video as a key frame [43]. Note that our key frame detection process might result in more than one key frame for a particular video. This is especially helpful in cases where the activities are complex or the events require more than one frame to describe the action. Our detected key frames are representative of the actions being performed in the video and hence can aid in automatic action detection/recognition. Example $f_{i,m}$ score outputs of our network and the corresponding ground truth obtained through LDA classifiers for two UCF101 videos are shown in Figure 4.

### 3.2. Implementation Details

We use the Caffe toolbox [65] for ConvNet implementation and all networks are trained on one NVIDIA GPU. Below are the implementation details of our technique:

**Feature Extraction for Frame-level Video Labelling.** In our experiments, we use the VGG-16 network [58] trained on the ImageNet dataset [66] to extract the appearance and motion features from the input video frames and their corresponding optical flow images. fc7-layer features of video frames and the optical flow images are extracted and concatenated into a 8192-dimensional visual feature vector. These features vectors are used to calculate the frame level scores $f_{i,m}$ using LDA and thus label/annotate them automatically.

**Network Architecture for Key Frame Detection.** Once again we employ the VGG-16 [58] model trained on the ImageNet [66] to design the two-stream ConvNets (Section 3.1). The two-stream network consists of appearance and motion networks which operate on RGB frames and dense optical frames respectively. These frames are calculated from two adjacent RGB frames. The input RGB image or optical flow frames are of size 256×340 pixels, and are center cropped to a size of 224×224 pixels. To fine-tune the network, we replace the fc6 layer with a concatenated layer, (which is obtained by aggregating the fc6 of appearance and motion ConvNets) and replace the fc7 with a 2-length layer. The softmax layer is replaced with a Euclidean loss layer. We use mini-batch stochastic gradient descent (SGD) to learn the network parameters, where the batch size is set to 16 and momentum is set to 0.9. We initialize the learning rate with $10^{-3}$ and decrease it by a factor of 10 for every 1600 iterations. We rescale the optical flow fields linearly to a range of [0, 255]. For the extraction of the optical flow images, we use the TVL1 optical flow algorithm [67] from the OpenCV toolbox. We use batch normalization.

**Fitting function setting:** In our experiments, we use the *smooth spline* function for fitting a curve using the predicted scores of each frame to automatically detect the key frames in a video. This function, has a parameter α that controls the fitting shape. We empirically set α=0.8 if the number of frames in a video are less than 60, α=0.6 if they are between 60 and 170, and α=0.1 if the number of frames are more than 170.

## 4. Experiments

This section gives the details of our experiments and presents the analysis.

### 4.1. Datasets

Literature is scarce on works performing automatic key frame annotation. In fact there are no benchmark datasets for key frame annotation in human action videos. To address this, we conduct experiments to automatically annotate key frames in human action videos in a dataset widely used for human action recognition, namely the UCF101 [20]. This dataset consists of 101 action classes with 13,320 video clips. It has at least 100 video clips for each action category. Besides its 101 categories, UCF101 has coarse definitions which divide the videos into human and object interaction, human and human interaction and sports. Although, this dataset is designed for human action recognition, we use it to perform key frame annotation and detection experiments.

For automatic key frame annotation and to train the key frame detection network we use 10 randomly chosen videos each from 20 classes, thereby making a total of 200 videos. To test the performance of our model, we randomly select 15 videos from each of these 20 classes (total 300 videos). Note that there is no overlap between training and test videos.

In order to verify our proposed key frame detection method, we also perform experiments on the video dataset VSUMM [21], which has been widely used to conduct video summarization based key frame detection experiments. This dataset includes 50 videos available online. The duration of the videos ranges from 1 to 10 minutes and the contents include cartoons, news and sports. Each of these have five human-created summaries, which are generated by five different subjects. Following the testing protocol set by [68,69], we also choose 20% videos for testing.

#### Evaluation Criteria

To demonstrate the efficacy of our approach, we compare it with several state-of-the-art approaches. (1) Traditional approaches [21,68,70,71]. (2) Deep learning approaches [4,72,73]. All the results are from their original papers or reported in [1,72].

To the best of our knowledge, literature is devoid of benchmarks on key frame annotation. There are indeed works that have used key frames (self defined) to detect actions but have not benchmarked the “accuracy” or “meaningfulness” of these key frames. This makes our task of comparing our results with other methods very challenging. Before we evaluate our experimental results, we need to define some meaningful evaluation metrics as our objective is to match the ground truth. One direct measure is to evaluate the number of key frames detected by our method as compared to the key frames annotated by the self supervised key frame annotation model. The second measure is to evaluate the temporal distance between the location of the detected key frames compared with the ground truth. To obtain this information for the test videos we separately pass them through our automatic key frame annotation model to get a sense of “ground truth” and then test them on our two-stream key frame detection network. Since we have both the number and location information of the key frames, in the following, we derive the two types of evaluation metrics accordingly.

**Key frame number match error:** Annotating too many irrelevant frames as key frames in a video would undermine the main objective of video summarization, action recognition. It will also affect subsequent processing in terms of more computations and less accuracy due to insignificant key frames. Thus, we propose a key frame number match error $E_{o}$ to evaluate the detection accuracy. We first assume the ground truth number of key frame for a given video is $Q_{o}$ and the predicted number of key frame is $P_{o}$, then, the number matching error can be simply described as:

(6) $$E_{o}=\left|P_{o}-Q_{o}\right|$$

**Key frame location match error:** The temporal location matching of key frames is very important in key frame detection, since it directly illustrates whether the annotated frame is indeed a key frame i.e., important for video summarization, action recognition and video retrieval. We assume that the ground truth key frame locations are $\left\{G_{l}^{\left(1\right)},G_{l}^{\left(2\right)},\cdots ,G_{l}^{\left(s\right)}\right\}$ and the predicted locations are $\left\{P_{l}^{\left(1\right)},P_{l}^{\left(2\right)},\cdots ,P_{l}^{\left(s\right)}\right\}$, then the location match error is described as:

(7) $$E_{l}=\pm \frac{1}{s}\sum_{x=1}^{s}\left|P_{l}^{\left(x\right)}-G_{l}^{\left(x\right)}\right|$$

Furthermore, we also consider F-Score, which is widely used in video summarization tasks, for evaluation [4,68,74], that is,

(8) $$P_{pd}=\frac{\#matchedframes}{\#framesinS_{1}},R_{pd}=\frac{\#matchedframes}{\#framesinS_{2}},F=\frac{2P_{pd}R_{pd}}{P_{pd}+R_{pd}}$$

where $S_{1}$ and $S_{2}$ are the generated summarized and ground-truth summarized frames. The final F-score is computed as *F*.

### 4.2. Results

(1) *Results on the UCF 101 Dataset*: Table 1 reports our two evaluation metrics for 20 action classes. It can be clearly seen that our method obtained low error for the number and location of key frames, and the average location error is less than one frame. Figure 4 visually depicts the number of key frames detected and their location as well as compares these metrics with the self supervised ground truth for all 20 classes given in Table 1.

**Figure 5:**
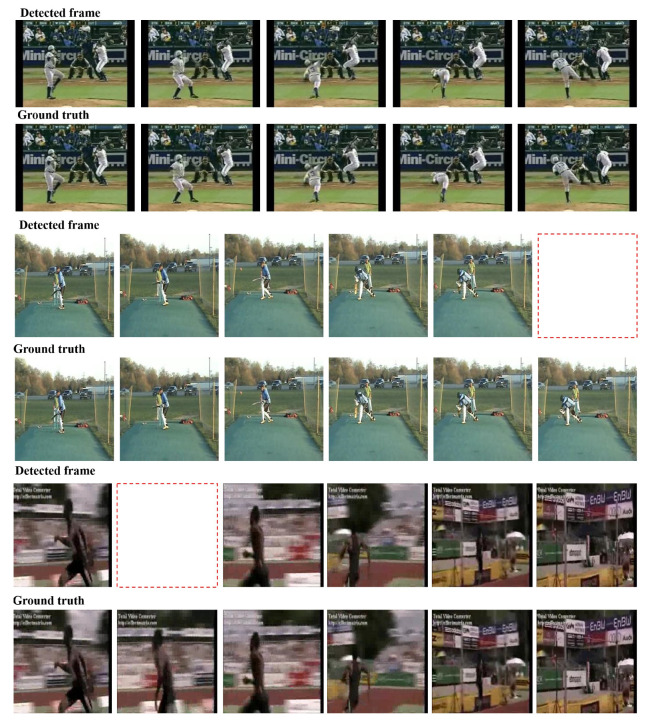
Examples of key frames detected along with the automatically annotated ground truth. Note that the empty red box denotes a missing key frame in the detected frames which implies that our method has detected more key frames than the automatically annotated ground truth. It can be observed that the key frame detected by our method are very similar to the ground truth. (best seen in colour).

Next we qualitatively report the output of our key frame annotation and detection models by visually analysing the detected key frames. Figure 5 shows the detected key frames compared with the self supervised automatically annotated ground truth for three classes. In the “Baseball Pitch” example, we observe that our deep model detects the key frames that seem to correspond to (i) the movement of lifting the leg, (ii) throwing the ball and (iii) the bending the body after the throw. In the “Cricket Shot” example, the detected key frames correspond to (i) being ready to bat, (ii) striking the ball with the bat. Similarly in the “High Jump” class, the annotated key frames correspond to (i) running, (ii) taking off, and (iii) jumping over the pole and landing. Such frame annotations resonate with previous works that have highlighted the general presence of three atomic actions in classes that can describe certain action [75].

Furthermore, from Figure 5, it can be observed that the key frames detected by our approach are align well with the ground truth. However, it seems that our two-stream ConvNet detects one more key frame in each of the first two depicted classes. In general, the set of key frames detected by our approach is very similar to the ground truth and can well represent the video content, which verifies the effectiveness of our approach. Moreover, these visualizations strengthen our claim that our approach is able to well and truly distinguish between human actions captured in video frames by detecting the differences in each frame. We further observe from these visualizations that our approach also implicitly learns to decompose actions from various classes into simpler sub-events. In other words, our approach can detect the key frames in videos and can correctly represent a video shot with less redundancy in frames.

(2) *Results on the VSUMM Dataset*: Table 2 compares our results with four traditional and three deep learning (usupervised and supervised) baselines on VSUMM [21]. We outperform the deep unsupervised learning based approach SUM-GAN [4] by an absolute gain of 9.6%. It is interesting to note that, compared to the deep supervised based approach [72], our method still obtains an absolute gain of 2.4%. The results demonstrate the efficacy of our method.

**Figure 6:**
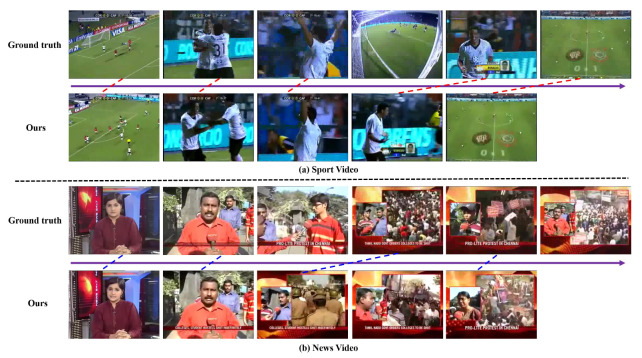
Examples of key frames detection of VSUMM dataset, along with the ground-truth key frames detection.

To better visualize the characteristics of our method, we presents two different kinds of key frame detection results using our method on the VSUMM dataset in Figure 6. It can be seen that although our method may not select exactly the same frame as the ground truth, our detected key frame has very good visual similarity with the ground truth and also looks reasonable. Especially, from Figure 6b, we can find that our method can also obtain relatively exact detection results very close to the ground truth. It is important to point out that we trained our deep network model without using any News videos. Thus, these result can verify the generalizability of our proposed method.

## 5. Conclusions

We presented a key frame detection deep ConvNets framework which can automatically annotate the key frames in human action videos. Such frames can correctly represent a human action shot with less redundancy, which is helpful for video analysis task such as video summarization and subsequent action recognition. The method used to address this problem learns to dynamically detect key frames of different videos. We trained training a deep ConvNet for key frame detection by combining the RGB video frames and their corresponding optical flow images. Since there is no benchmark dataset for evaluating key frame detection, we used the popular action recognition dataset UCF101 to perform our experiments. However, this dataset lacks frame-level labels. To mitigate this issue, we employed a self supervised learning method to automatically annotate frame-level labels by combining the CNN features and LDA. We verified our method on the UCF101 dataset and obtained encouraging results. Furthermore, to demonstrate the efficacy of our network trained on dataset UCF101, we also performed experiments on the video dataset VSUMM and obtained state-of-the-art results. To the best of our knowledge, this work is the first to report results for automatic key frame annotation in human action videos via deep learning.

## Figures and Tables

**Table 1:** Our technique can detect more than one key frame in a video. The table shows the difference in the number of key frames detected versus the automatically annotated ground truth as well as the position of these key frames relative to the ground truth in 20 classes of the UCF101 dataset.

Average Error in Key Frame Detection		
Class Name	Number Detected	Key Frame Position
Baseball Pitch	±1.73	±0.455
Basket. Dunk	±1.64	±0.640
Billiards	±2.18	±1.300
Clean and Jerk	±2.27	±1.202
Cliff Diving	±2.45	±0.627
Cricket Bowl.	±2.45	±1.14
Cricket Shot	±1.27	±0.828
Diving	±2.00	±0.907
Frisbee Catch	±1.73	±0.546
Golf Swing	±2.45	±0.752
Hamm. Throw	±1.73	±1.223
High Jump	±1.73	±0.434
Javelin Throw	±2.45	±0.555
Long Jump	±2.27	±0.611
Pole Vault	±2.64	±1.139
Shotput	±2.00	±0.564
Soccer Penalty	±2.09	±0.712
Tennis Swing	±1.64	±0.554
Throw Discus	±2.09	±0.642
Volley. Spike	±1.54	±0.633
Average accuracy	±2.02	±0.773

**Table 2:** Comparison of our proposed key frame detection approach compared to several state of the art (F-score%).

Method	F-Score%
VSUMM. [21]	67
DDC [70]	71
Gong et al. [68]	60.3
Zhang et al. [71]	61.0
SUM-GAN [4]	62.5
Fu et al. [72]	69.7
AVS [73]	66.2
Ours	72.1
